# Construction of a quaternary ammonium salt platform with different alkyl groups for antibacterial and biosensor applications

**DOI:** 10.1039/c7ra11001d

**Published:** 2018-01-12

**Authors:** Xue Lv, Chuang Liu, Shixin Song, Yun Qiao, Yuanjiao Hu, Pengfei Li, Zhaokun Li, Shulin Sun

**Affiliations:** Changchun University of Technology Changchun 130012 China sunshulin1976@163.com +86-431-8571-6467; Beijing Academy of Printing & Packaging Industrial Technology, Beijing Institute of Graphic Communication Beijing 102600 China

## Abstract

An electrochemical platform was designed using biocompatible quaternary ammonium salts containing alkyl groups with different chain lengths as electrode materials for visible protein immobilization on a glassy carbon (GC) electrode. The electrode was constructed using a simple self-assembly method relying on the electrostatic interaction between negatively charged hemoglobin (Hb) and positively charged quaternary ammonium materials. The Hb/quaternary ammonium salts/GC assembly exhibited excellent catalytic and electrochemical activities. Additionally, the structure–function properties of the quaternary ammonium salts on the electrochemical behavior of Hb was systematically investigated for various alkyl chain lengths between monomer and polymeric structures. Meanwhile, the corresponding bactericidal activities of the monomers and related polymers were evaluated by determining the minimum bactericidal concentration (MBC), minimum inhibitory concentration (MIC), and inhibitory zone diameters against bacteria. The results of these studies demonstrated that the quaternary ammonium monomers not only immobilized more proteins, but also displayed better antibacterial activity as alkyl chain length increased. Moreover, polymers possessed higher antimicrobial activities than their monomeric counterparts. However, the efficiency of the direct electron transfer process and the antibacterial properties of long-chain polymers were limited because they were prone to aggregation and blistering. In summary, the present results provide convenient access to direct electrochemistry using an immobilized redox protein. Furthermore, the potential to use the obtained materials in the construction of third-generation electrochemical biosensors was evaluated.

## Abstract 2

The immobilized-protein in an appropriate alkyl chain length of DM displayed higher antibacterial activities, exhibited a more effective DET and a promising catalytic activity toward H_2_O_2_.

## Graphical abstract

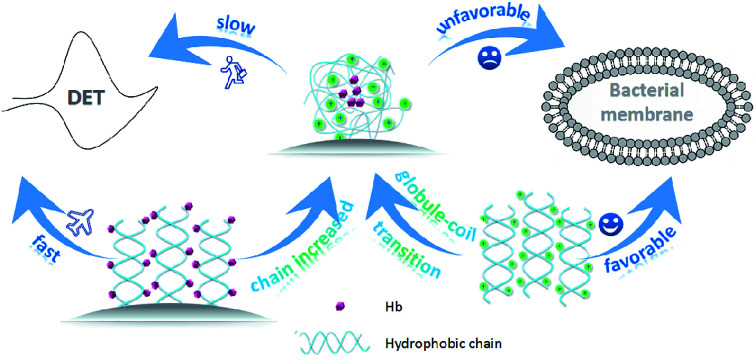

## Introduction

The electron transfer abilities of redox proteins on the surface of working electrodes have drawn considerable attention. Such research offers insight toward an understanding of the signal transmission mechanisms in biological systems. In addition, it provides a foundation for constructing novel sensitive biosensors. However, the absorptive denaturation and low diffusion of proteins on bare electrodes block the direct electron-transfer (DET) process. Thus, many researchers have endeavored to enhance direct electrochemical reactions and retain bioactivities on electrodes by choosing biocompatible electrode materials and suitable methods of protein immobilization.^1–3^ Several synthetic materials have been used to immobilize proteins for constructing electrochemical biosensors, including insoluble surfactants, hydrogel polymers, and biopolymers.^4,5^

Antibacterial agents have drawn increasing attention because of their high antibacterial efficacy and chemical stability.^6–9^ In general, the most common antimicrobial reagents (oxidants and electrophilic agents^10,11^) are highly toxic and harmful to the environment. Recently, cationic polymers containing quaternary ammonium salt groups have been identified as desirable antimicrobial materials^12,13^ due to their low toxicity and eco-friendliness. The Nguyen research group^14^ evaluated the bactericidal activity of four quaternary ammonium salts by determining their minimum bactericidal concentrations (MBCs). Jiang^15^ and coworkers studied the antibacterial activity of dimethylaminoethyl methacrylate (DM) on cotton fiber surfaces. The results of their experiments showed that a DM/cotton fiber copolymer with various alkyl segments exhibited promising antibacterial activity.

To date, the exact mechanism for the observed antibacterial activity has not been elucidated, although several researchers have devoted a great deal of effort to the endeavor. Roy *et al.*^16^ found that copolymers quaternized with octyl bromide was effective against *E. coli*. However, Thorsterinsson *et al.*^17^ synthesized several polymeric quaternary ammonium compounds (PQAC)s, and found that alkyl chain length might have an inverse relationship with antibacterial activity. Lu *et al.*^18^ prepared a series of quaternary ammonium monomers and their homopolymers. Interestingly, they discovered that the antibacterial activity of the polymers with four-membered alkyl chains were more effective. Tiller *et al.*^19^ discovered that short-chain length poly(vinyl pyridine) was more effective than its long-chain length counterpart. Kanazawa *et al.*^20–23^ found that the antibacterial activities of cationic salts were strongly affected by spacer length and molecular structure, and their activity increased as spacer length increased. A consensus has not yet reached surrounding the role of the alkyl substituents in the efficacy of antibacterial agents. It is well-understood that the mechanisms of antibacterial activity vary, even within single antibacterial systems. Unfortunately, insufficient quantitative data are presently available, and the mechanisms are complicated in ways we cannot yet predict. Further study is needed to understand the alkyl chain effect.

Previous research has shown that quaternary ammonium salt monomers with various chain lengths can be synthesized and processed. In particular, Zhou *et al.*^24^ considered DM as a suitable biomaterial for performing direct electrochemistry of proteins because its characteristics^25–27^ such as its biocompatibility,^28–30^ high hydrophilicity, non-toxicity, and low cost. In addition, there are some reports in the literature in which DM can catalyze redox processes, creating a base for future electrochemical sensors.

To date, little is known about the role of the alkyl chain in enhancing the transmission of biological signals of proteins during immobilization. Therefore, DM was chosen as a model. We set out to prepare a series of new quaternary ammonium monomers with chain length between 2 and 12 and their respective polymers, and reveal the effect of the chain length on anti-bacterial and electrochemical behaviors.

Herein, Hb was taken as a model protein and combined with quaternary ammonium salt to create a novel platform for the study of the electron-transfer process. Hb was immobilized on modified electrodes under a favorable environment for undergoing a self-assembly method. Then, to clarify the structural features that enable electron transmission, DMs with different alkyl chain were used as reference electrodes. The results of this systematic investigation indicate that optimizing chain length could help researchers immobilize more proteins and efficiently accelerate signal transduction. Hence, they are expected to be useful for manipulating interfacial properties to adapt the DET of Hb and fabricate novel bioelectronic devices based on quaternary ammonium salts.

## Experimental

### Materials

Hemoglobin (Hb) was purchased from Sigma Chemical Co. DM, was purchased from Shanghai Chemical Co., Ltd. Bromo ethane, butane bromide, bromo hexane, decane bromide, azobisisobutyronitrile and potassium ferricyanide (K_3_Fe(CN)_6_) were purchased from National Pharmaceutical Group Chemical reagent Co., Ltd. Acetone, acetonitrile and LB medium were purchased from Tianjin Chemical Reagent Factory. The concentration of phosphate-buffered saline (PBS) solution was 0.1 M and the pH was 7.0. Chitosan, potato dextrose agar (potato dextrose agar, PDA) and MH agar (MHA) were purchased from Difco company. All solutions were prepared using Milli-Q purified water (18.0 MΩ cm^−1^). Above agents were analytical grade and used without further purification.

### Synthesis of monomers

**Fig. 1:**
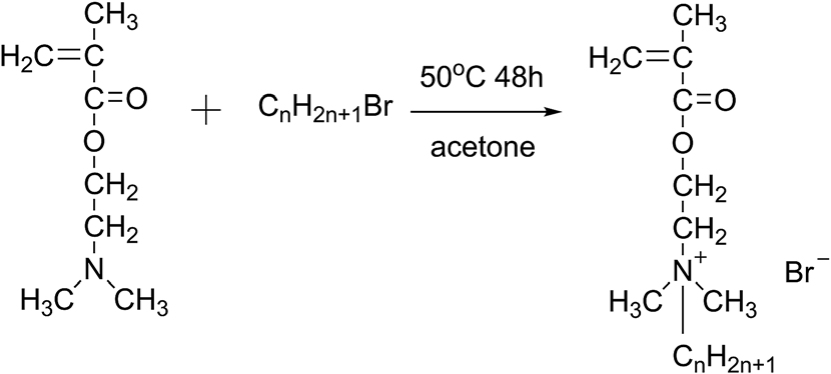
The schematic of preparing of quaternary ammonium salt monomer (C_*n*_H_2*n*+1_Br, *n* = 2/DM-EB, *n* = 6/DM-HB, *n* = 10/DM-DB, *n* = 12/DM-LB).

A mixture of DM and alkyl bromides in a molar ratio of 1 : 1.2 was added to a three-necked round bottom flask equipped with a stirrer, a cooler, and a thermometer, using acetone as the reaction medium. The mixture was stirred at 50 °C for 48 h. Finally, white needle-shaped crystals were obtained by cooling and filtering the reaction solution, then washing with ether several times and drying under vacuum at 35 °C.^20^ The obtained monomers were namely DM-EB (yield 90%), DM-HB (yield 85%), DM-DB (yield 80%) and DM-LB (yield 73%) respectively. The progression of the synthesis was shown in Fig. 1.

### Synthesis of polymeric quaternary ammonium salts

**Fig. 2:**
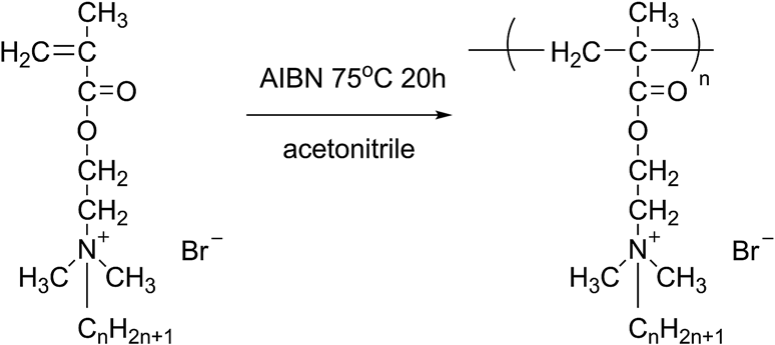
The schematic of preparing of polymeric quaternary ammonium salts.

All polymerizations were carried out in a 250 mL three-neck flask equipped with a condenser, a magnetic stirrer and purged with nitrogen for several minutes, the desired amounts of monomer, initiator and solvent were added into the flask to react for 20 h under a nitrogen atmosphere. Finally, the synthesized homopolymers were immersed in a large excess of acetone. After that, the eventual product was dried under vacuum at 50 °C for 48 h. The yields of polymeric quaternary ammonium salts (Fig. 2) were 51%, 65%, 67% and 69% for poly(DM-EB), poly(DM-HB), poly(DM-DB) and poly(DM-LB) respectively.

### Construction of film electrodes

Firstly, glassy carbon electrode (GCE) were polished with 1.0, 0.3, 0.05 μm alumina slurry, then rinsed with ultrapure water, and sonicated in acetone, ethanol and deionized double-distilled water sequentially. To prepare the Hb/DM-monomer film and the corresponding Hb/polymer film-modified GCE, suspensions of monomers and their corresponding polymers were prepared by mixing DM-monomer (1 mg mL^−1^, solvent: water) and related polymer (1 mg mL^−1^, solvent: methanol) with Hb (0.5 mL, 0.5 mM) in PBS. The prepared dispersion (5 μL) was deposited onto the surface of a pretreated GCE. According to Song's work,^5^ chitosan sol (8 μL, 1 wt%) was added to encapsulate the GCE. The dried uniform film-modified GCE was stored at 4 °C. Here, the driving force of adsorption between Hb and DM-modified GCE were electrostatic and van der Waals interactions. For comparison, Hb/GCE, DM-monomer/GCE were prepared by similar procedures as described above.

### Strains

*Escherichia coli* (*E. coli*, ATCC25922), *Staphylococcus aureus* (*S. aureus*, ATCC6538), *Candida albicans* (*C. albicans*, ATCC 14053) and *Aspergillus fumigates* (*A. fumigates*, Af293) were purchased from the institute of microbiology, Chinese Academy of Sciences, Beijing, PR China (CAS standard).

## Measurements and apparatus

### Instruments

Ultraviolet-visible (UV-vis) absorption spectra were measured by a UV-2450 spectrophotometer (Shimadzu, Japan). Fourier transform infrared (FT-IR) spectra were recorded by Avatar-360 spectrophotometer (USA) in the range from 400–4000 cm^−1^ with scan rate of 60 times. ^1^H nuclear magnetic resonance (^1^H-NMR) spectra were recorded on a Bruker AV II-400 MHz spectrometer (Bruker, Switzerland) using CDCl_3_ as solvent. The samples (50 mg) were put into an NMR tube, then a solution of CDCl_3_ (5 mL) was injected into the NMR tube, then the tube was shaken until the solids were dissolved in CDCl_3_. The test were performed at room temperature and TMS was applied. The corresponding peaks at 0 ppm in the ^1^H-NMR was ascribed to TMS.

Elemental analysis measurements were performed on Elementar Vario MICRO CUBE (Elementar Analysensysteme GmbH, USA). HEAL FORCE biological safety cabinets were conducted on HF SAFE-1200 of Hong Kong. YJ-876 type clean bench and MJX-250-IIB microcomputer control mold incubator were purchased from Suzhou Antai air technology Co., Ltd. and Nikon YS2-H based bio-microscope company, respectively.

Cyclic voltammetry were performed on an electrochemical workstation CHI 660C. The experiment of electrochemical used a three electrode system comprising a Pt wire as auxiliary electrode, a saturated calomel electrode as reference and film-modified electrode as working electrode. All solutions were conducted under a N_2_ atmosphere during the electrochemical experiments.

### Bacteria and growth condition

*Escherichia coli* (ATCC25922), *Staphylococcus aureus* (ATCC6538) were inoculated into LB medium. *Candida albicans* (ATCC 14053) and *Aspergillus fumigates* (Af 293) were inoculated into PDA medium. They were chosen as testing microorganisms and streaked out on nutrient agar plates, with incubated at 37 °C for 24 h.

### Inhibition zone measurement

In order to visualize the antibacterial effect of the monomers and related polymers, the inhibition zone was measured. The bacterial concentration was adjusted to 108 CFU mL^−1^, and the solution was placed on the surface of an agar plate. The suspension of the tested bacteria was coated on the surface of the medium, then 60 μL of each tested antibacterial agent samples (10 mg mL^−1^) were respectively filled into the circle (3 mm diameter). The corresponding number represent: N, nothing; 1, sterile water; 2, DMSO; 3, ethanol; 4, DM-EB; 5, DM-HB; 6, DM-DB; 7, DM-LB; 8, poly(DM-EB); 9, poly(DM-HB); 10, poly(DM-DB); 11, poly(DM-LB). Samples were placed on the inoculated agar plates and incubated overnight at 37 °C. Digital images of the plates were captured to measure the inhibition zone diameter. Each experiment was repeated twice, with three replicates each time. The results of drug sensitivity criteria are shown in Table 1.

**Table 1:** The criteria of drug sensitivity

Bacteriostatic ring diameter (mm)	Sensitivity
>20	Extremely
15–20	High
10–15	Medium
<10	Low
0	No

### Minimum bactericidal concentration (MBC) and minimum inhibitory concentration (MIC) measurement

MBC was measured to determine bactericidal activity. The antimicrobial tests were conducted as follows: the synthesized quaternary ammonium monomers and polymers were dissolved in saline and maintained at a concentration of 10 mg mL^−1^ by several two-fold dilutions using sterile water. The concentration varied from 10 to 1.2 × 10^−3^ mg mL^−1^. Then, each solution was mixed with an equal volume of a bacterial suspension, and mixed at 36 °C in a shaking water bath overnight. The MBC value was determined by finding the concentration at which no bacteria survived on the agar plate. Each experiment contained three replicates and was repeated twice.

Minimum inhibitory concentration (MIC) values were determined to monitor the activity of the antimicrobial agents. Each of the samples were dissolved in saline and diluted by half in sterile distilled water before testing. Subsequently, the lowest concentration of inhibition of the visible growth of a microorganism was considered the MIC. The samples were stored after overnight incubation.

## Results and discussion

### IR analysis

**Fig. 3:**
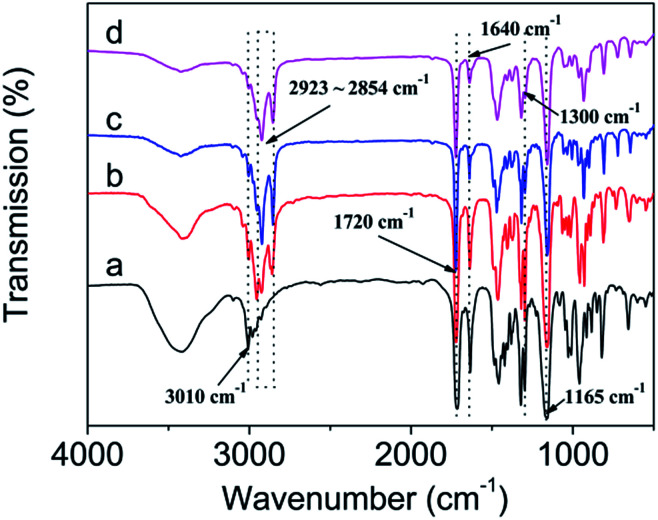
FTIR spectra of DM-EB (a), DM-HB (b), DM-DB (c) and DM-LB (d).

As shown in Fig. 3, the chemical structure of DM-EB (a), DM-HB (b), DM-DB (c), and DM-LB (d) were characterized by IR spectroscopy.^4^ The strong peak at 3010 cm^−1^ was attribute to the stretching vibration of =C–H in curve a–d, the absorbance at 1720 cm^−1^ can provide information about the structure of –C=O–. The peak at 1640 cm^−1^ corresponds to the shear-vibration of pendent double bonds and the peak at 2923–2854 cm^−1^ which belonged to the asymmetric stretching vibration of –CH_3_ and –CH_2_– group, the position of the absorption peak at 1165, 1300 cm^−1^ was attributed to the structure of –C–O– and –C–N–. All above illustrated that four monomers were successfully synthesized.

**Fig. 4:**
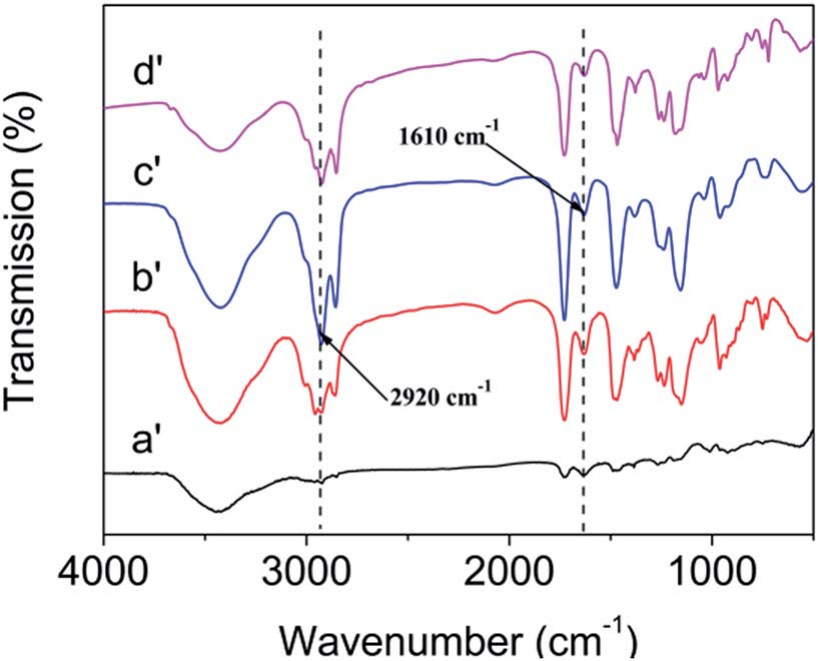
FTIR spectra of poly(DM-EB) (a′), poly(DM-HB) (b′), poly(DM-DB) (c′) and poly(DM-LB) (d′).

As shown in Fig. 4, the chemical structure of poly(DM-EB) (a′), poly(DM-HB) (b′), poly(DM-DB) (c′), and poly(DM-LB) (d′) were characterized by IR spectroscopy.^4^ Obviously, the corresponding strong peak of monomer at 3010 cm^−1^ (=C–H) and 1640 cm^−1^ was disappeared in polymer. All above illustrated that four polymers were successfully synthesized.

### NMR analysis

**Fig. 5:**
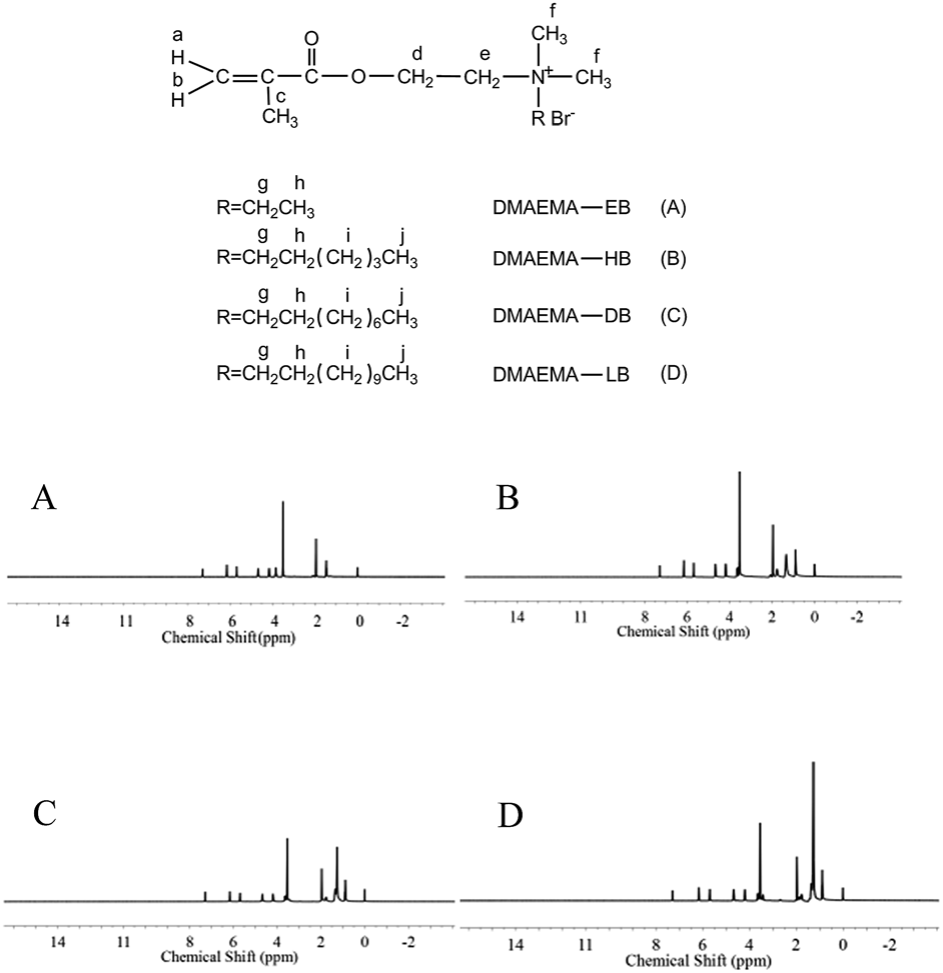
^1^H-NMR spectra of quaternary ammonium monomers.

^1^H-NMR tests were performed to confirm the formation of quaternary ammonium monomers, and the data are displayed in Fig. 5. All of the protons can be attributed to the expected signals. DM-EB (Fig. 5A), H NMR (CDCl_3_, *δ*): 6.13 (1H, a), 5.64 (1H, b), 1.95 (3H, c), 4.65 (2H, d), 4.15 (2H, e), 3.50 (6H, f), 3.83 (2H, g), 1.45 (3H, h). DM-HB (Fig. 5B), H NMR (CDCl_3_, *δ*): 6.15 (1H, a), 5.68 (1H, b), 1.96 (3H, c), 4.67 (2H, d), 4.17 (2H, e), 3.53 (6H, f), 3.61 (2H, g), 1.76 (3H, h), 1.31 (6H, i), 0.89 (3H, j). DM-DB (Fig. 5C), H NMR (CDCl_3_, *δ*): 6.16 (1H, a), 5.69 (1H, b), 1.97 (3H, c), 4.67 (2H, d), 4.18 (2H, e), 3.54 (6H, f), 3.61 (2H, g), 1.77 (3H, h), 1.26–1.35 (12H, i), 0.89 (3H, j). DM-LB (Fig. 5D), H NMR (CDCl_3_, *δ*): 6.16 (1H, a), 5.69 (1H, b), 1.97 (3H, c), 4.67 (2H, d), 4.19 (2H, e), 3.54 (6H, f), 3.61 (2H, g), 1.77 (3H, h), 1.26–1.67 (18H, i), 0.87 (3H, j).

### Elemental analysis

The elemental analysis data of four quaternary ammonium monomer were showed in Table 2. The results of elemental analysis exhibited that the experimental value (actual) was basically consistent with the theoretical value (calc.). Based on the data above, we have synthesized the quaternary ammonium monomers with different alkyl chain successfully.

**Table 2:** Elemental analysis results of quaternary ammonium salts

Sample	C%		H%		N%	
	Calc.	Actual	Calc.	Actual	Calc.	Actual
DM-EB	0.4508	0.4466	0.07138	0.07266	0.04840	0.04845
DM-HB	0.5213	0.5190	0.08378	0.08368	0.04344	0.04345
DM-DB	0.5708	0.5695	0.09249	0.09282	0.0370	0.03650
DM-LB	0.5905	0.5893	0.09795	0.09764	0.0344	0.03425

### UV-visible spectroscopic analysis

**Fig. 6:**
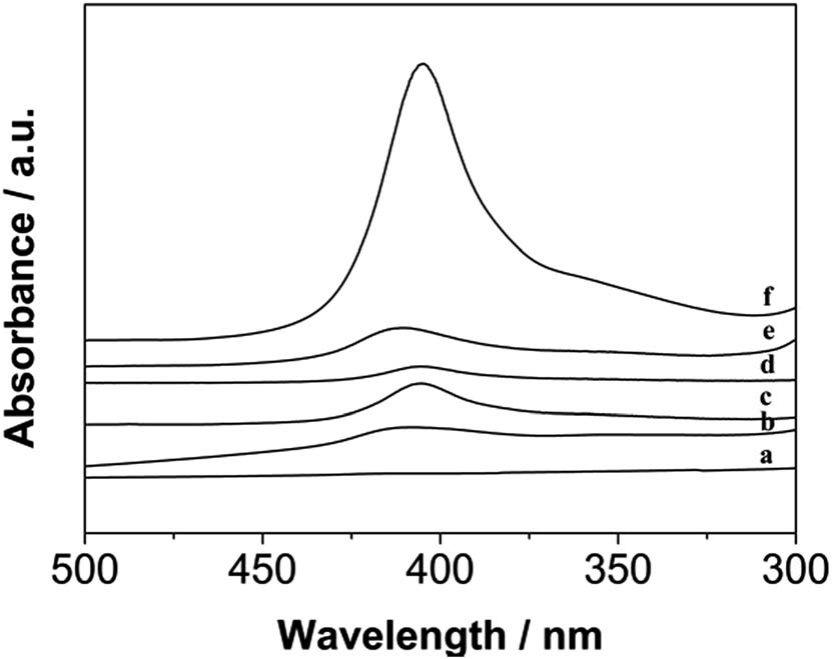
UV-vis absorption spectra of DM-EB (a), DM-EB/Hb (b), DM-HB/Hb (c), DM-DB/Hb (d), DM-LB/Hb (e) and Hb (f) in 0.1 M PBS solution.

As shown, based on the view of biotechnology application, the position of Soret absorption band is critical to evaluate the immobilization of Hb on DM film.^4^ As well known, we performed UV-vis test to observe the absorption spectra between free Hb and DM film/Hb composite. Obviously to discover that DM film/Hb exhibited a strong Soret band with an absorption maximum at 408 nm (curve b–e, Fig. 6), which was close to that of free aqueous Hb (curve f, Fig. 6), with absorption maximum at 407 nm. However, the DM-EB had no obvious adsorption peak (curve a, Fig. 6) in the wavelength region. The results indicated that the microenvironment around the Hb pocket was no denaturation during immobilization on the electrodes.

### Electrochemical redox behavior of protein-modified electrodes

**Fig. 7:**
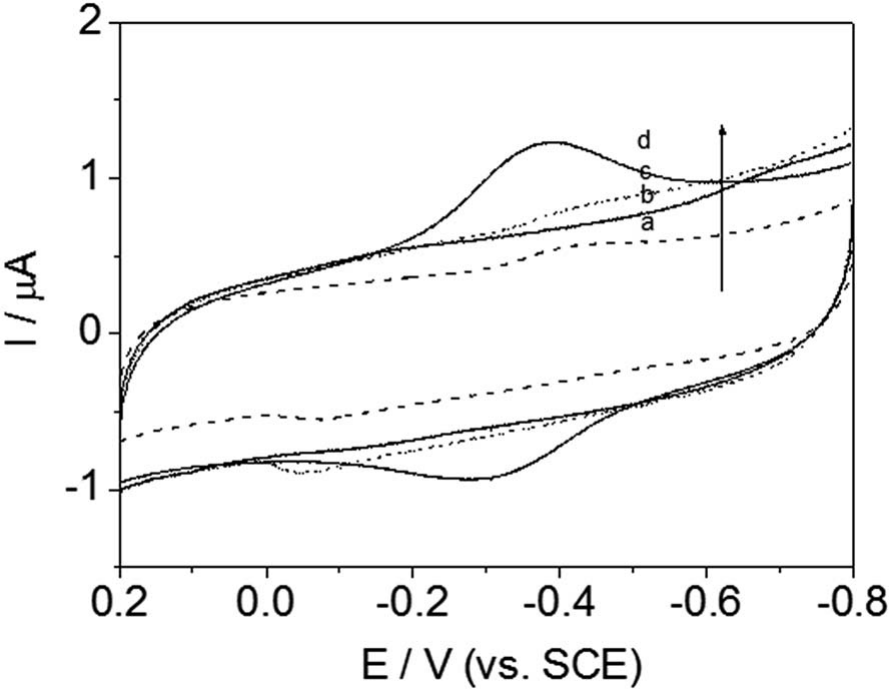
Cyclic voltammograms of DM-EB/glassy carbon electrode (GCE) (dashed line), Hb/GCE (solid line b), bare electrode (dotted line) and DM-EB/Hb/GCE (solid line d) in PBS at pH 7.0. Scanning rate, 200 mV s^−1^.

Cyclic voltammetry (CV) was always used to investigate the electrochemical behavior of modified electrodes, especially signal transmission of immobilized redox protein. For curve (a–c) in Fig. 7, no clear redox peak was observed at GCE modified with those materials, without Hb, at a scanning rate of 200 mV s^−1^, indicating that the DM-EB, Hb and GCE were not electroactive in the potential range from −0.8 to 0.2 V.

However, in contrast, a significantly different electrochemical response was observed for DM-EB electrodes after modification with Hb. A pair of well defined and nearly symmetrical redox peaks was observed obviously (curve d, Fig. 7), which attributing the protein assembled on the surface of DM-EB/GCE remained its catalytic activity and demonstrated that direct electron transfer between Fe^III^/Fe^II^ center of immobilized Hb had taken place. The cathodic peak potential (*E*_pc_) and anodic peak potential (*E*_pa_) of DM-EB/Hb/GCE were located at −0.3940 V and −0.3108 V, respectively. The peak potential separation of 84 mV, suggested that the immobilized protein underwent a quasi-reversible electrochemical reaction. The formal potential (*E*_p_), defined as the average of *E*_pa_ and *E*_pc_, was −0.3524 V. The data presented herein demonstrates that DM-EB can provide an efficient pathway for the electron transfer of Hb and a suitable microenvironment and orientation for the immobilization of Hb, which maintained its original catalytic activity.

**Fig. 8:**
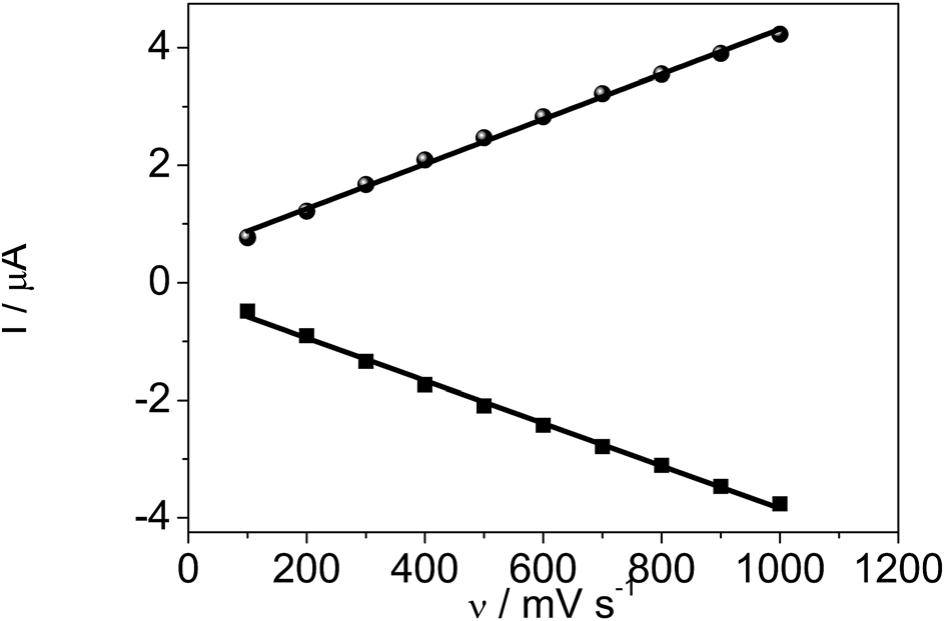
Plots of oxidation peak current, *I*_a_ (●) and reduction peak current, *I*_c_ (■) *vs.* scanning rates for DM-EB/Hb/GCE.

The influence of scan rates on the voltammetric responses of the DM-EB/Hb/GC electrode were also investigated (Fig. 8). The anodic (*I*_a_) and cathodic (*I*_c_) peak currents both rose linearly with the increase in scan rate from 100 to 1000 mV s^−1^, indicating that the redox reaction of proteins on the DM-EB-modified electrode was a surface-controlled process. According to Faraday's law, *Q* = nFAΓ* (where *F* is the Faraday constant, Γ* represents the surface concentration of electroactive Hb, *Q* is calculated by integrating the Hb reduction peak, *n* presents the number of electrons transferred, and *A* is the area of electrode (0.07 cm^2^)). In this case, the calculated Γ* for DM-EB/Hb/GC electrode was 4.542 × 10^−11^ mol cm^−2^. Generally, the traditional theoretical monolayer coverage for Hb on electrode surface was 2 × 10^−11^ mol cm^−2^, which indicated that Hb was effectively immobilized on the surface and the Hb entrapped in the matrix was in favorable orientation.

### Effect of chain length on electrochemical behavior

**Fig. 9:**
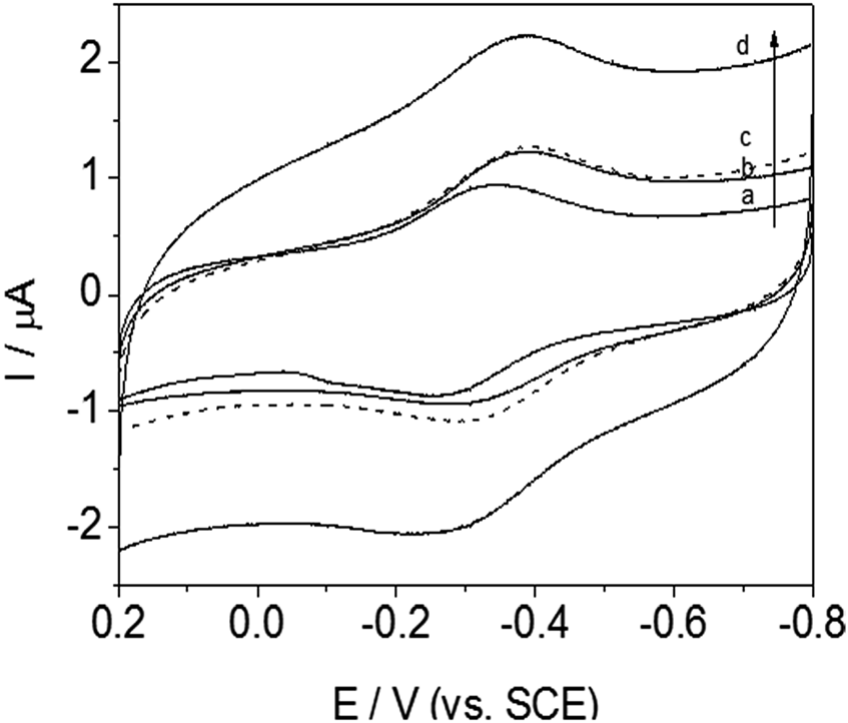
Cyclic voltammograms of DM-LB/Hb/GCE (solid line, a), DM-EB/Hb/GCE (solid line, b), DM-HB/Hb/GCE (dashed line, c) and DM-DB/Hb/GCE (solid line, d) in PBS (pH 7.0). Scanning rate, 200 mV s^−1^.

**Fig. 10:**
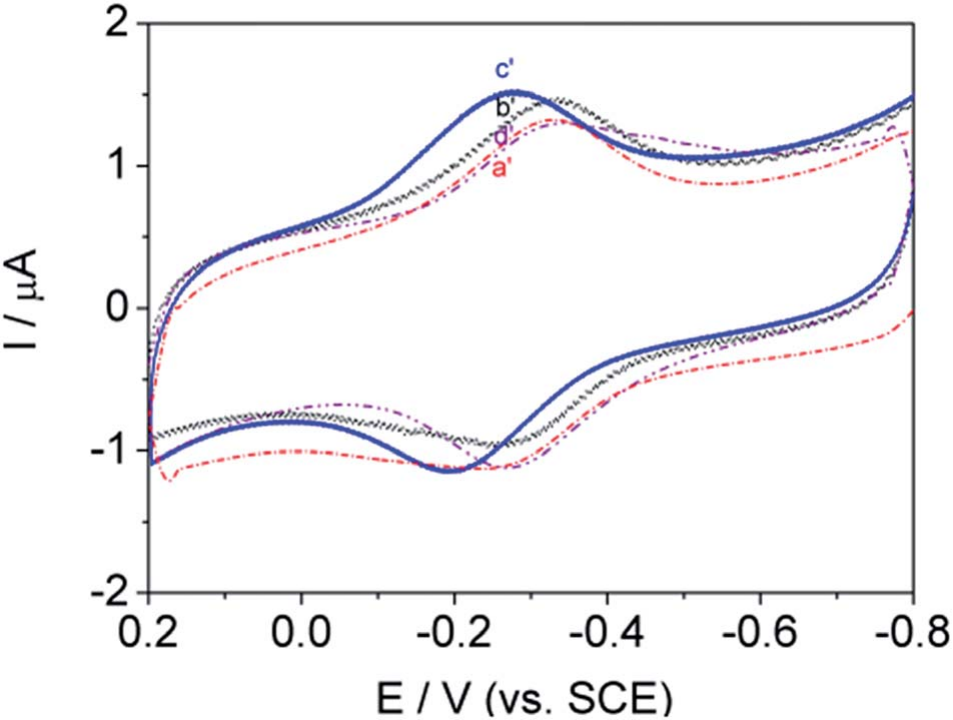
Cyclic voltammograms of poly(DM-LB)/Hb/GCE (red dashed line, a′), poly(DM-EB)/Hb/GCE (black line, b′), poly(DM-HB)/Hb/GCE (blue line, c′) and poly(DM-DB)/Hb/GCE (purple dotted line, d′) in PBS (pH 7.0). Scanning rate, 200 mV s^−1^.

Most protein studies have focused on eliminating absorption and denaturation on electrodes and identifying various classes of biocompatible materials. This work was designed to identify the chain length structural features that enable signal transmission and understand the influence of adsorption behavior on the electrode. Herein, a systematic study of the alkyl chain length of DM-monomer and polymer-modified GC electrodes has been conducted, successfully enabling DET from Hb to the electrode. In attempting to identify promising lengths of alkyl segments, four antibacterial monomers and related polymers, namely, DM-LB (a), poly(DM-LB) (a′), DM-EB (b), poly(DM-EB) (b′), DM-HB (c), poly(DM-HB) (c′), and DM-DB (d), poly(DM-DB) (d′) respectively, were all available for protein immobilization (Fig. 9 and 10). Somewhat surprisingly, an interesting phenomenon was observed that all electrodes exhibited similar curves. This finding supports our understanding that these modified electrodes provide a compatible soft interface with biomolecules to support the electron transfer process.

Nevertheless, from the CV curve (a to d) obtained for DM-modified GCE in Fig. 9, we found that redox peak currents was somewhat different. Among this series of Hb/film modified GCE, it was noted that redox peak currents of curve d (DM-DB) were the largest than the others (curve a–c, for DM-LB, DM-EB, DM-HB respectively).

According to the tunneling effect proposed by Liu,^5^ the longer the length of the molecular chains, the slower the electron transfer rate of electrode-fixed Hb. However, increases in the length of the alkyl segment enhanced the electron transfer rate (*k*_s_) and *E*_p_ (Table 3). Usually, the trace with a more positive *E*_p_ corresponds with favorably oriented adsorbed Hb. The opposite phenomenon occurred during this investigation; this was attributed to strong physical interactions including hydrogen bonding between DM-modified films and the –O– and –NH– groups on the protein. The monomer molecules interact with the electrode surface in a specific manner, aligning and anchoring the protein molecules in a suitable orientation. The hydrophobicity of the monomer (rather than its charge) was found to be crucial in promoting the response of these proteins on glassy carbon electrodes. As a result, the DM-LB-modified GCE possessed an outstanding *E*_p_, showing that Hb partially intercalated into the space of matrix, shortening the distance between GC and Hb.

**Table 3:** Electrochemical parameters of Hb in various length modified electrode

Electrodes	*E*_pc_/V	*E*_pa_/V	*E*_p_/V	Δ*E*_p_/mV	*k*_s_/s	*Γ* × (10^−11^ mol cm^−2^)
DM-EB/Hb/GCE	−0.3940	−0.3108	−0.3524	83.2	0.650	4.542
DM-HB/Hb/GCE	−0.3892	−0.3084	−0.3488	80.8	0.640	4.598
DM-DB/Hb/GCE	−0.3509	−0.2709	−0.3109	80.0	0.634	7.073
DM-LB/Hb/GCE	−0.3460	−0.2580	−0.3020	88.0	0.697	3.568
Poly(DM-EB)/Hb/GCE	−0.3428	−0.2530	−0.3000	89.8	0.711	6.076
Poly(DM-HB)/Hb/GCE	0.2800	−0.1910	−0.2355	89.0	0.705	9.336
Poly(DM-DB)/Hb/GCE	−0.3590	−0.2688	−0.3139	90.2	0.714	5.374
Poly(DM-LB)/Hb/GCE	−0.3210	−0.2300	−0.5510	91.0	0.720	4.093

As shown in Fig. 10, the influence of polymers on biological signal transmission was very interesting. With increasing alkyl-chain length, redox peak currents increased between curve b′ and c′. In contrast, the redox peak currents decreased with further increases in alkyl chain length (curve d′–a′). This revealed that the polymer molecules tended to aggregate and blister easily so that a considerable number of active groups were not able to make contact with the protein (curve d′–a′).

Unfortunately, this hinders mechanistically driven designs for even more effective surface-active polymers. These results suggest that the direct electron transfer of polymer salts decreased as the side-chain length increased.

As shown in Table 3, with the increasing number of alkyl groups, the values of *k*_s_ were slightly different using Laviron's equation, meaning that the tunneling distance of Hb with each of the four electrodes was similar. Another intriguing feature was found: the surface coverage (Γ*) of Hb immobilized on DM-DB GCE was larger than the others, which may be because the strong positive charge of the quaternary ammonium monomer facilitates further self-assembly with the oppositely charged protein by electrostatic interactions. Nevertheless, long alkyl chain length resulted in irregular arrangement and limited movement of the coiled molecular chains covering the electrode. Furthermore, the length of chain is critical to the Γ* of the entrapped Hb on matrix-modified electrodes.

Importantly, the *k*_s_ and Γ* of polymers were larger than those of their corresponding monomers. Because polyelectrolytes were combine the merits of polymers and electrolytes, and the polymers have a higher charge density than that of the precursory monomer due to their higher molecular weights, they exhibit a stronger direct electron transmission.

### Electrochemical catalysis of H_2_O_2_ by DM-EB/Hb/GCE

**Fig. 11:**
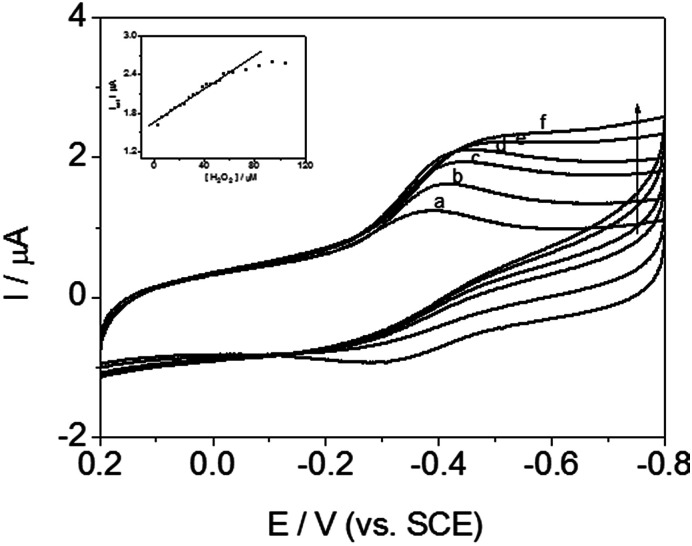
Cyclic voltammograms of DM-EB/Hb/GCE with (a) 0 μmol L^−1^, (b) 3.5 μmol L^−1^, (c) 24.5 μmol L^−1^, (d) 31.5 μmol L^−1^, (e) 63.0 μmol L^−1^ and (f) 84.0 μmol L^−1^ H_2_O_2_ in PBS (pH 7.0), and plots of the electrocatalytic current (*I*_cat_) *vs.* H_2_O_2_ concentration on DM-EB/Hb/GCE (inset). Scanning rate, 200 mV s^−1^.

Cyclic voltammetric measurement of DM-EB/Hb/GCE were carried out to detected H_2_O_2_ for evaluating the catalytic activity of immobilized Hb. As illustrated in Fig. 11, the reduction peak current at −0.35 V (*vs.* SCE) enhanced significantly with increasing the concentration of H_2_O_2_ (curve b–f), which indicated that Hb immobilized on the electrode exhibited high bioelectrocatalytic activity.

In addition, the inset in Fig. 11 showed that the reduction peak current obtained increased linearly with H_2_O_2_ concentration over the range of 3.5–63.0 μM. The linear regression equation was *y* = 1.64147 + 0.01346*x*, *R* = 0.987 (*y* means the peak current, *x* is the concentration of H_2_O_2_), LOD = 1.17 μM (S/N = 3). Based on the discussion above, the signal transmission behavior indicated that the present modified electrode successfully detected H_2_O_2_. The electrocatalytic activity of mechanism was expressed by the following reaction:

1 HbFe(iii) + H^+^ + e^−^ → HbHFe(ii)

2 2HbHFe(ii) + H_2_O_2_ → 2HbFe(iii) + 2H_2_O

What's more, DM-LB/Hb/GCE, DM-HB/Hb/GCE and DM-DB/Hb/GCE, similar catalytic behaviors and calibration curves were observed, which were attributed to their structural similarity and intrinsic catalytic activities to peroxide compounds. Herein, for the other three electrodes (DM-HB/Hb/GCE, DM-DB/Hb/GCE, DM-LB/Hb/GCE), the corresponding equation was *y* = 1.43762 + 0.01355*x*, *R* = 0.96; *y* = 2.27987 + 0.01419*x*, *R* = 0.97; *y* = 0.99496 + 0.01954*x*, *R* = 0.98 respectively.

### Inhibition zone measurement

**Fig. 12:**
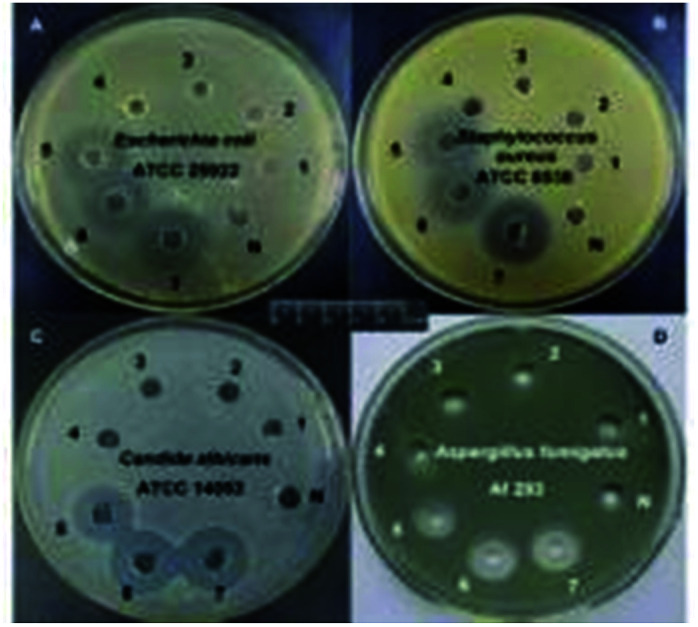
The antibacterial activity of quaternary ammonium monomer of (A) (ATCC25922), (B) (ATCC 6538), (C) (ATCC 14053) and (D) (Af 293). Namely, no. N is blank for comparison; no. 1–3 represent sterile water, DMSO, ethanol respectively; no. 4 is DM-EB; no. 5 is DM-HB; no. 6 is DM-DB; no. 7 is DM-LB.

Inhibition zone measurement was carried out to test the quaternary ammonium monomer's antibacterial abilities. Interesting, a distinct phenomenon was observed (Fig. 12 and Table 4). Namely, no. 1–3 exhibited no obvious inhibition halo. However, no. 4–7 all formed an inhibition zone against *E. coli*, *S. aureus*, *C. albicans*, and *A. fumigates*, demonstrating the antibacterial activity of the DM-monomer. No. 8–11 show the antibacterial activity of the corresponding polymers under the same conditions.

**Table 4:** The inhibitory zone diameter of quaternary ammonium monomers and related polymers (10 mg mL^−1^)

Strain	The diameter of bacteriostatic circle (mm) (*n* = 5)										
	1	2	3	4	5	6	7	8	9	10	11
*E. coli*	0.00	0.00	0.00	0.00	23.95^a^	24.57^a^	26.01^a^	13.45^c^	9.21^d^	0.00	0.00
*S. aureus*	0.00	0.00	0.00	8.44^d^	21.44^a^	24.37^a^	28.54^a^	12.10^c^	11.42^c^	0.00	0.00
*C. albicans*	0.00	0.00	0.00	0.00	21.19^a^	24.31^a^	25.02^a^	13.34^c^	9.14^d^	0.00	0.00
*A. fumigates*	0.00	0.00	0.00	8.94^d^	14.53^c^	18.71^b^	19.86^b^	9.16^d^	8.28^d^	0.00	0.00

a Bacteriostatic ring diameter (mm) ≥ 20.

b 20 > Bacteriostatic ring diameter (mm) ≥ 15.

c 15 > Bacteriostatic ring diameter (mm) ≥ 10.

d Bacteriostatic ring diameter (mm) < 10.

As shown in Table 4, increasing length of alkyl group increased the diameter of the inhibition zone diameter. This indicated that increasing the chain length improved diffusion and bacteriostatic ability.

Conversely, the inhibition zone diameter became smaller as the chain increased in the related polymers. Even for 10 and 12-membered alkyl chains, no inhibition zone could be detected.

Long alkyl chains may bend and therefore “bury” the positively charged groups, limiting electrostatic interactions and lowering antibacterial potency.

### MBC and MIC test

The effects of varying the chain length on new quaternary ammonium monomers and related polymers were investigated systematically by MBC and MIC. The experimental results are shown in Tables 5 and 6. Short-chained monomers had high MBC values. Conversely, long chained monomers had low MBC values. The same variation in MIC values is demonstrated in Table 6. The results were consistent with previously reported findings.^31,32^ These phenomena were explained by a widely accepted mechanism.^33,34^ For small cationic antibacterial agents, long alkyl chains favor strong reactions with the cytoplasmic membranes of bacteria because the hydrophobic interaction between the lipid layer of the cell wall and the side chain is enhanced in longer chain lengths, improving antibacterial activity.

**Table 5:** MBC values of quaternary ammonium salts monomers and related polymers

Sample	DM-EB		DM-HB		DM-DB		DM-LB	
	Monomer (mg mL^−1^)	Polymer (mg mL^−1^)	Monomer (μg mL^−1^)	Polymer (mg mL^−1^)	Monomer (μg mL^−1^)	Polymer (mg mL^−1^)	Monomer (μg mL^−1^)	Polymer (mg mL^−1^)
*E. coli*	>10	0.625	39.1	2.5	19.5	>10	4.9	>10
*S. aureus*	5	1.25	78.1	1.25	19.5	>10	2.4	>10
*C. albicans*	>10	0.625	78.1	2.5	19.5	>10	9.8	>10
*A. fumigates*	2.5	2.5	312.5	5	15.6	>10	15.6	>10

**Table 6:** MIC values of quaternary ammonium salts monomers and related polymers

Sample	DM-EB		DM-HB		DM-DB		DM-LB	
	Monomer (mg mL^−1^)	Polymer (mg mL^−1^)	Monomer (μg mL^−1^)	Polymer (mg mL^−1^)	Monomer (μg mL^−1^)	Polymer (mg mL^−1^)	Monomer (μg mL^−1^)	Polymer (mg mL^−1^)
*E. coli*	—	0.1	6.3	0.4	3.2	—	1.6	—
*S. aureus*	0.4	0.2	12.5	0.2	3.2	—	0.8	—
*C. albicans*	—	0.1	12.5	0.4	3.2	—	1.6	—
*A. fumigates*	0.4	0.4	50	0.4	25	—	25	—

Compared with their corresponding monomers, polymers tend to exhibit higher bactericidal activities because their higher charge density increases the efficiency of absorption by the negatively charged bacterial surface (Table 5).

However, these results exhibited that two of the polymers had decreased bactericidal activity compared to their monomers (Tables 5 and 6). The phenomenon could be interpreted as follows: with increasing chain length, the polymers easily tangled and contracted. In addition, the combined inter- and intra-molecular hydrophobic attractions could be greater than the repulsive force of the positive charges in the chain. This increases their tendency to aggregate and form a sphere, rendering them inaccessible to bacterial cells and therefore weakening their antibacterial activity.

## Conclusions

The electrochemical performances of Hb immobilized on a series of quaternary ammonium monomer and polymers with different alkyl lengths were studied for the first time. The resulting Hb/DM-film/GC modified electrode exhibited promising catalytic activity toward H_2_O_2_ with a low detection limit, demonstrating that DM films could serve as a soft interface and stable adsorption state for Hb. More interestingly, Hb/DM-modified electrodes exhibited a more effective DET and more favorable orientation as the length of the alkyl segment was increased. In addition, the regularity of antibacterial activities were in conformance with the results of electrochemical experiments. Taken together, these findings provide a useful model of quaternary ammonium monomers as promising biomimetic materials with excellent properties and provide a novel platform for the fabrication of biosensors and biomolecular electronic devices.

## Conflicts of interest

There are no conflicts to declare.

## Supplementary Material
